# Evaluating the impact of a trial of labor after cesarean section on labor duration: a retrospective cohort study

**DOI:** 10.1186/s12884-024-06744-0

**Published:** 2024-08-15

**Authors:** Hikaru Ooba, Jota Maki, Hisashi Masuyama

**Affiliations:** Department of Obstetrics and Gynecology, Okayama University Hospital, 2-5-1 Shikata-cho, Kita-Ku, Okayama City, 700-8558 Okayama Prefecture Japan

**Keywords:** Labor duration, Trial of labor after cesarean section, Vaginal birth, Cesarean section, Propensity scores, IPTW

## Abstract

**Background:**

Cesarean section (C-section) rates are increasing globally, and repeated C-sections are associated with increased maternal morbidity. Trial of labor after C-section (TOLAC) is an approach to reduce the recurrence of C-sections. However, limited research exists on the impact of cesarean scars on labor duration in TOLAC, considering the termination of labor through C-section and selection bias. This study aimed to investigate the impact of cesarean scars on labor duration in TOLAC participants, accounting for potential confounding factors and biases.

**Methods:**

This retrospective cohort study included 2,964 women who attempted vaginal birth at a single center in Japan from 2012 to 2021. The study categorized participants into TOLAC (*n* = 187) and non-TOLAC (*n* = 2,777) groups. Propensity scores were calculated based on 14 factors that could influence labor duration, and inverse probability of treatment weighting (IPTW) was applied. Cox proportional hazards regression analysis estimated hazard ratios (HRs) for labor duration, with and without IPTW adjustment. Sensitivity analyses used propensity score matching, bootstrapping, and interval censoring to address potential biases, including recall bias in the reported onset of labor.

**Results:**

The unadjusted HR for labor duration in the TOLAC group compared to the non-TOLAC group was 0.83 (95% CI: 0.70–0.98, *P* = 0.027), indicating a longer labor duration in the TOLAC group. After adjusting for confounding factors using IPTW, the HR was 0.98 (95% CI: 0.74–1.30, *P* = 0.91), suggesting no significant difference in labor duration between the groups. Sensitivity analyses using propensity score matching, bootstrapping, and interval censoring yielded consistent results. These findings suggested that the apparent association between TOLAC and longer labor duration was because of confounding factors rather than TOLAC itself.

**Conclusions:**

After adjusting for confounding factors and addressing potential biases, cesarean scars had a limited impact on labor duration in TOLAC participants. Maternal and fetal characteristics may have a more substantial influence on labor duration.

**Supplementary Information:**

The online version contains supplementary material available at 10.1186/s12884-024-06744-0.

## Introduction

The prevalence of cesarean section (C-section) is on the rise globally [1], with some nations reporting that up to 50% of births are conducted via C-Sect. [2]. In Japan, the rate has increased to over 18% [3], and tertiary care facilities indicate that 37.3% of childbirths are C-Sect. [4]. Such repeated procedures are associated with extended surgical durations, increased risk of severe adhesions, higher blood loss, and elevated transfusion needs [5]. Furthermore, post-operation women may face complications such as infertility, high-risk subsequent pregnancies, postpartum menorrhagia, and dysmenorrhea [6]. These symptoms threaten their quality of life.

Trial of labor after C-section (TOLAC) offers a promising approach to reduce the frequency of repeat cesarean births and their adverse effects [7]. Successful vaginal birth after C-section (VBAC) [8] not only presents lower infection, fever, and postpartum hemorrhage risks but also proves to be more cost-efficient than elective repeat cesarean delivery (ERCD) [9]. While successful TOLAC is associated with the least maternal morbidity, the hazards of failed TOLAC surpass those of planned repeat C-Sect. [10]. Factors such as occiput-posterior fetal position, extended second labor stage, maternal age, and large-for-gestational-age fetuses contribute to TOLAC failure [11]. The primary concern with unsuccessful TOLAC is uterine rupture, which prompts many hospitals to proceed cautiously with TOLAC attempts [12]. Additionally, the duration of labor, especially the length of the second stage, is a critical risk factor for uterine rupture during TOLAC [13].

Research on labor duration in women with previous C-sections remains limited. A study focusing on cervical dilation time during the first labor stage [7] reported median durations of 3.0 h for TOLAC and 2.8 h for non-TOLAC participants. However, factors such as maternal age and parity influence labor duration, rendering simple time comparisons potentially biased. Previous investigations of TOLAC labor duration have often overlooked cesarean terminations and failed to adjust for variables affecting labor time. Our study aimed to reveal the impact of cesarean scars on the duration of vaginal labor with these limitations.

## Methods

### Study design

This retrospective cohort study utilized data from a single medical center from January 1, 2012, to December 31, 2021. In the institution, women with a prior C-section were provided with written information about the risks and benefits of TOLAC and ERCD during antenatal visits before the onset of labor. Informed consent was obtained regarding the chosen mode of delivery. Women who opt for TOLAC were also informed about the possibility of requiring an emergency C-section during labor, based on the same criteria as for women without a prior C-section. The decision to attempt TOLAC or undergo ERCD was made by the women themselves, and those who choose TOLAC received the same standard of care as women undergoing a normal vaginal birth. Labor induction was not routinely performed for women undergoing TOLAC unless deemed necessary by the attending obstetrician. We included singleton pregnancies between 37 weeks 0 days and 41 weeks 6 days, both spontaneous and induced labor. The exclusion criteria were elective C-sections, preterm births before 37 weeks, post-term births after 42 weeks, intrauterine fetal demise (IUFD), and multiple gestations. Comprehensive information on maternal background, medical history, delivery details, and postnatal and neonatal care was obtained from electronic health records. Data were extracted according to predefined common categories provided by the Japan Society of Obstetrics and Gynecology Perinatal Database. Participants who underwent vaginal birth were categorized into TOLAC and non-TOLAC groups based on their history of C-section. Participants who required emergency C-section during the vaginal birth trial were censored. Labor duration was defined as the total time from labor onset to delivery, encompassing both the first and second stages of labor. The onset of labor was determined based on the participants’ self-reports.

### Statistical analysis

We assumed an effect size of 0.20, alpha error of 0.05, beta error of 0.20, and dropout rate of 5%. Based on the 2021 birth statistics of the facility, we presumed the proportion of TOLAC to be 0.07, resulting in a calculated sample size of 3,007 participants. Influenced by prior studies [9, 14–18], we identified 14 factors potentially affecting labor duration. These factors included maternal age, Body Mass Index (BMI), maternal nationality, history of vaginal birth, pre-pregnancy smoking habits, gestational diabetes, premature rupture of membranes, fetal sex, birth weight, fetal position, induction intended to promote labor, labor analgesia, uterine fundal pressure, and vacuum-assisted delivery. These factors were used to calculate propensity scores. The distributions of these variables are presented in Table 1. For each factor, continuous variables were analyzed using the t-test, while categorical variables were examined using the chi-square test. The Standardized Mean Difference (SMD) [19] was also calculated. Data with missing information on delivery time or with more than 25% missing values for any item were excluded. Given that the database was regularly updated by medical staff immediately after childbirth, missing information was assumed to be missing at random (MAR) [20]. Multiple imputations were employed to address the missing values. Subsequently, logistic regression was used to calculate propensity scores based on the 14 factors above. The area under the Receiver Operating Characteristic curve (ROC-AUC) of the propensity scores was computed. For the TOLAC group, weights were determined as the inverse of the propensity score, while for the control group, weights were the inverse of one minus the propensity score, calculating the inverse probability of treatment weighting (IPTW) [21]. These weights were then applied to the dataset. To address the increased variance in estimates due to propensity scores being close to zero or one, we trimmed the top and bottom 1% of the propensity scores. Survival curves for each labor duration were created, from which labor duration curves were depicted, and hazard ratios were estimated using Cox proportional hazards regression analysis. Statistical analyses were conducted using R software (version 4.2.3, R Foundation for Statistical Computing, Vienna, Austria).

**Table 1:** Participant background

Characteristic	*N*	Non-TOLAC *N* = 2,777^1^	TOLAC *N* = 187^1^	SMD^2^	95% CI^23^	*p*-value^4^
**Maternal age**	2,964			-0.41	-0.56, -0.26	< 0.001
Mean (SD)		31 (5)	33 (5)			
**Maternal body mass index**	2,914			-0.13	-0.28, 0.02	0.077
Mean (SD)		25.4 (3.6)	25.9 (3.4)			
Unknown		46	4			
**Maternal country of origin**	2,964			0.10	-0.04, 0.25	0.2
Japanese		2,626 (95%)	172 (92%)			
Other country		151 (5.4%)	15 (8.0%)			
**History of vaginal birth**	2,964			0.53	0.38, 0.68	< 0.001
( - )		1,490 (54%)	146 (78%)			
( + )		1,287 (46%)	41 (22%)			
**Smoking**	2,037			0.04	-0.14, 0.22	0.8
( - )		1,611 (84%)	108 (83%)			
( + )		296 (16%)	22 (17%)			
Unknown		870	57			
**Gestational diabetes mellitus**	2,964			0.23	0.08, 0.38	< 0.001
( - )		2,586 (93%)	161 (86%)			
( + )		191 (6.9%)	26 (14%)			
**Premature rupture of membranes**	2,964			0.04	-0.11, 0.19	0.7
( - )		1,895 (68%)	131 (70%)			
( + )		882 (32%)	56 (30%)			
**Fetal sex**	2,964			0.04	-0.11, 0.19	0.6
Female		1,364 (49%)	88 (47%)			
Male		1,413 (51%)	99 (53%)			
**Fetal birth weight**	2,964			0.01	-0.13, 0.16	0.9
Mean (SD)		3,097 (382)	3,091 (440)			
**Fetal position**	2,961			0.10	-0.05, 0.25	0.4
Cephalic position		2,730 (98%)	186 (99%)			
breech presentation		44 (1.6%)	1 (0.5%)			
Unknown		3	0			
**Labor induction**	2,956			0.50	0.36, 0.65	< 0.001
( - )		2,260 (82%)	181 (97%)			
( + )		509 (18%)	6 (3.2%)			
Unknown		8	0			
**Labor analgesia**	2,964			0.04	-0.11, 0.19	0.7
( - )		2,582 (93%)	172 (92%)			
( + )		195 (7.0%)	15 (8.0%)			
**Vacuum-assisted delivery**	2,811			0.24	0.07, 0.41	0.003
( - )		2,441 (91%)	118 (84%)			
( + )		229 (8.6%)	23 (16%)			
Unknown		107	46			
**Uterine fundal pressure**	2,963			0.40	0.25, 0.55	< 0.001
( - )		2,570 (93%)	187 (100%)			
( + )		206 (7.4%)	0 (0%)			
Unknown		1	0			
**Censor**	2,964			0.62	0.47, 0.77	< 0.001
( - )		2,670 (96%)	141 (75%)			
( + )		107 (3.9%)	46 (25%)			
**Duration of delivery**	2,964			0.07	-0.08, 0.21	0.4
Mean (SD)		614 (563)	576 (597)			

^1^n (%)

^2^Standardized Mean Difference

^3^CI = Confidence Interval

^4^Welch Two Sample t-test; Pearson’s Chi-squared test

### Outcome

The primary outcome was designated as the hazard ratio for labor duration based on the presence of cesarean scars following the application of IPTW. The secondary outcome was determined as the hazard ratio for labor duration associated with cesarean scars without the application of IPTW.

### Sensitivity analysis

IPTW estimates the average treatment effect (ATE) across the entire trial population, including participants with and without cesarean scars. However, extreme propensity scores can lead to unstable estimates [22]. Therefore, as a sensitivity analysis, we conducted an assessment using propensity score matching. By matching participants with similar propensity scores from both the exposed and control groups, the distribution of covariates in the matched subset became closer to that in the exposed study population. Propensity score matching and IPTW have different assumptions and limitations, allowing for the strengthening of result robustness by examining the effects in both populations.

Considering the potential dependency of the results on a specific dataset, a sensitivity analysis was conducted using the bootstrap method. The bootstrap algorithm can be used to align the values of the explanatory variables with those of a given target distribution [23]. We randomly resampled the original dataset to generate bootstrap samples. For each sample, Cox proportional hazard models were applied both with and without IPTW to calculate hazard ratios. The distribution of hazard ratios was estimated from the obtained samples. This process was repeated 1,000 times.

To address potential recall bias in the reported onset times of labor, we performed a sensitivity analysis using interval censoring [24]. Interval censoring is a method that allows for uncertainty in the exact timing of an event, such as the onset of labor, by using an interval within which the event is known to have occurred. Using interval censoring, we aimed to assess the robustness of our findings to potential inaccuracies in the reported onset times of labor. The lower bound of the interval was defined as the reported onset time of contractions minus a specified duration, while the upper bound was set as the reported onset time plus the specified duration. We considered three different durations: 4, 8, and 12 h, to account for varying degrees of potential recall bias. In cases where the lower bound of the interval was negative, it was rounded to 0.1 to ensure non-negative survival times. Using the obtained interval-censored data, we estimated the HRs and their 95% CIs for the association between TOLAC and the duration of labor. The analyses were conducted both with and without IPTW to account for potential confounding factors.

## Results

**Fig. 1:**
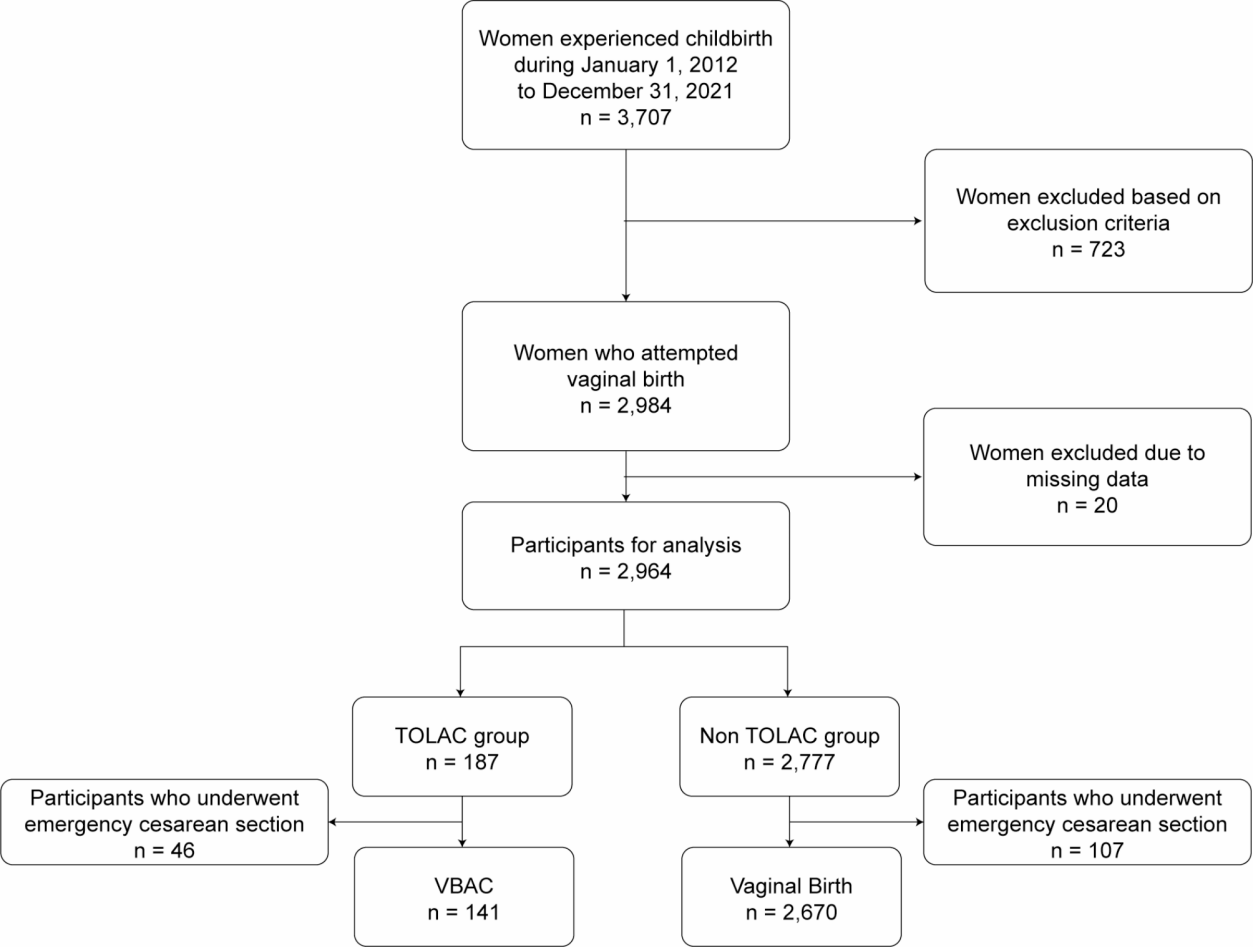
Flowchart of the study population. TOLAC: trial of labor after cesarean section; VBAC: vaginal birth after cesarean section

**Fig. 2:**
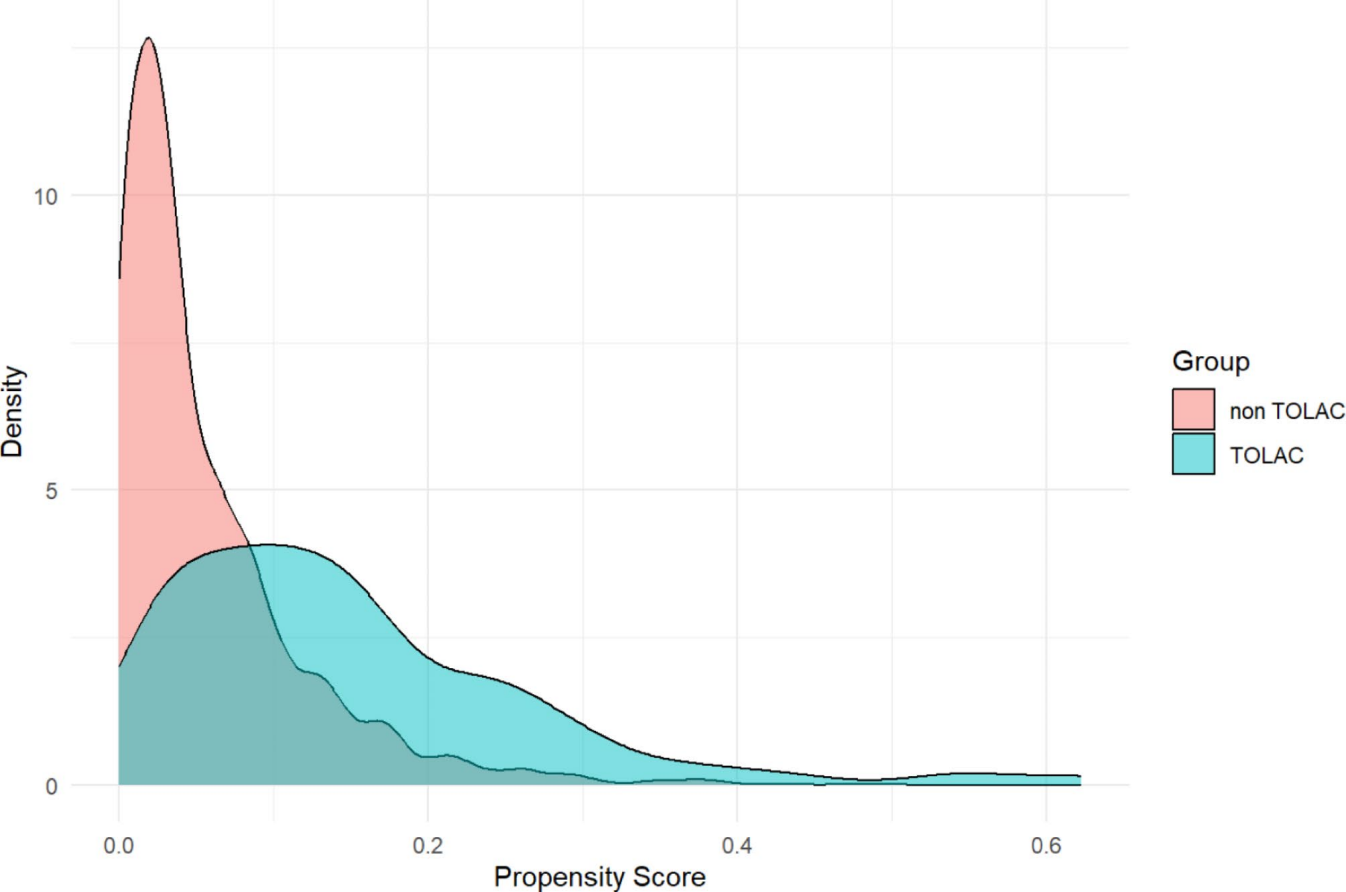
Density plot of propensity scores for the trial of labor after cesarean section (TOLAC, green) and non-TOLAC (red) groups

During the observation period, 3,707 women gave birth. A total of 723 women were excluded due to elective C-section (*n* = 582), multiple gestations (*n* = 91), preterm births before 37 weeks (*n* = 141), post-term births after 42 weeks (*n* = 4), and IUFD (*n* = 73). Of the remaining 2,984 women who attempted vaginal birth, 20 were excluded due to incomplete data on labor duration, resulting in 2,964 participants being included in the analysis. The non-TOLAC group consisted of 2,777 women (93.7%), whereas the TOLAC group included 187 women (6.3%). During the observation period, 46 individuals (25.4%) in the TOLAC group and 107 individuals (3.9%) in the non-TOLAC group were censored because of emergency C-sections. Figure 1 illustrates the recruitment process. Overall, compared to the non-TOLAC group, the TOLAC group included women of older age, and had a higher prevalence of gestational diabetes, vacuum-assisted delivery, and emergency C-section. Women with a history of vaginal birth, labor induction, and uterine fundal pressure were more common in the non-TOLAC group. The variable for maternal smoking was excluded due to more than 25% missing data. The characteristics of the study population are presented in Table 1. The ROC-AUC of the propensity score was 0.80 (95% CI: 0.77–0.84). Figure 2 summarizes the probability density of the propensity scores for TOLAC and non-TOLAC women. As expected, the distribution of propensity scores for the TOLAC group shifted towards 1, while that for the non-TOLAC group shifted towards 0.

**Fig. 3:**
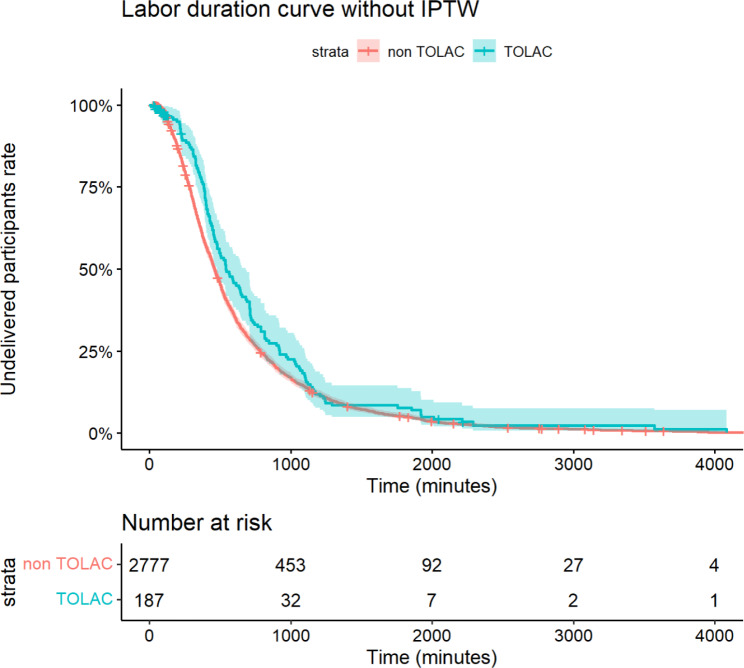
Labor duration curves for the trial of labor after cesarean section (TOLAC, green) and non-TOLAC (red) groups without inverse probability of treatment weighting (IPTW). Shaded areas represent 95% confidence intervals

**Fig. 4:**
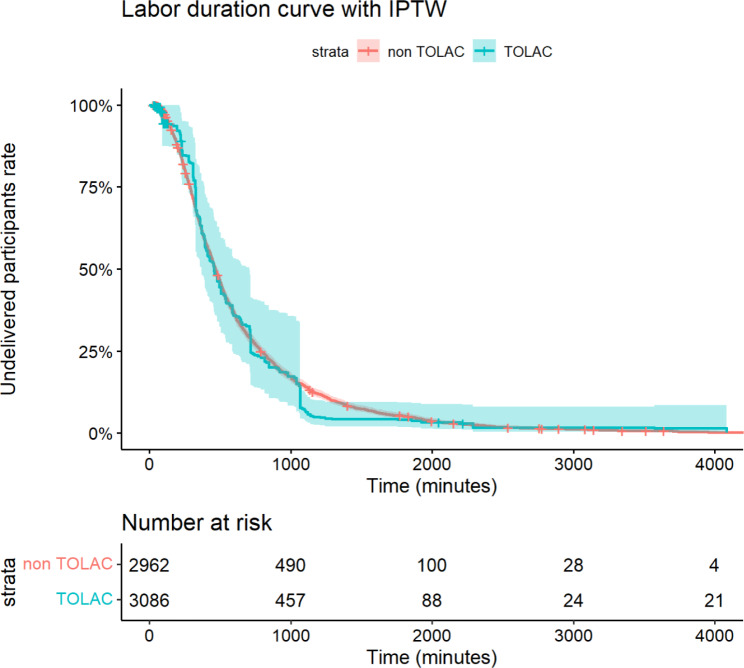
Labor duration curves for the trial of labor after cesarean section (TOLAC, green) and non-TOLAC (red) groups with inverse probability of treatment weighting (IPTW). Shaded areas represent 95% confidence intervals

Figure 3 displays the labor duration curves for participants stratified by TOLAC without IPTW, along with the 95% CI. The Log-Rank test yielded a result of *P* = 0.03. According to the Cox proportional hazards analysis, the HR for TOLAC was 0.83 (95% CI: 0.70–0.98, *P* = 0.027), indicating a longer labor duration in the TOLAC group when not adjusting for confounding factors. Figure 4 shows the labor duration curves for participants stratified by TOLAC with IPTW. The Log-Rank test resulted in *P* = 0.70. The Cox proportional hazards analysis revealed an HR for TOLAC of 0.98 (95% CI: 0.74–1.30, *P* = 0.91), suggesting no significant difference in labor duration between the TOLAC and non-TOLAC groups after adjusting for confounding factors using IPTW.

The results of the sensitivity analyses are presented in Supplementary Tables 1 and 2. The propensity score matching analysis yielded an HR of 1.02 (95% CI: 0.81–1.28, *P* = 0.88), which was consistent with the main analysis using IPTW (HR 0.98, 95% CI: 0.74–1.30, *P* = 0.91). The bootstrap analysis also produced similar results, with an HR of 0.83 (95% CI: 0.70–0.97) without IPTW and 1.07 (95% CI: 0.87–1.33) with IPTW. The interval censoring analysis (Supplementary Table 2) showed that the HRs for TOLAC decreased as the interval range increased when not adjusting for confounding factors (± 4 h: HR 0.68, 95% CI: 0.48–0.97, *P*=0.031; ±8 h: HR 0.45, 95% CI: 0.24–0.83, *P* = 0.011; ±12 h: HR 0.40, 95% CI: 0.20–0.79, *P* = 0.008). However, after adjusting for confounding factors using IPTW, the HRs were closer to 1 and not statistically significant (± 4 h: HR 1.00, 95% CI: 0.90–1.11, *P* = 0.96; ±8 h: HR 1.05, 95% CI: 0.87–1.27, *P* = 0.63; ±12 h: HR 1.23, 95% CI: 0.99–1.51, *P* = 0.06). These findings suggest that the observed association between TOLAC and labor duration is robust to potential recall bias in the reported onset of labor, particularly after adjusting for confounding factors.

## Discussion

When not adjusting for covariates through IPTW, the duration of labor was significantly longer for those undergoing TOLAC than for those who did not (HR = 0.83, 95% CI: 0.70–0.98, *P* = 0.027). After applying IPTW to account for potential confounders, the difference in labor duration between the two groups was not significant (HR = 0.98, 95% CI: 0.74–1.30, *P* = 0.91). These findings suggested that the apparent association between TOLAC and longer labor duration was because of confounding factors rather than TOLAC itself. These trends remained consistent even after conducting sensitivity analyses using propensity scores and the bootstrap method. It also suggested that the observed relationship is robust even after accounting for potential recall bias in the reported start time of delivery.

As emphasized in prior research [25, 26], the most significant predictor of successful TOLAC is a history of vaginal birth. The likelihood of a successful VBAC increases if there is a previous history of vaginal birth, previous successful TOLAC, or if natural labor commences during a current pregnancy with attempted TOLAC [27]. Studies examining the duration of TOLAC labor stratified based on the history of vaginal birth [28] indicated that TOLAC groups without a history of vaginal birth were comparable to nulliparous control groups. Conversely, the TOLAC groups with a history of vaginal birth were similar to the multiparous control groups, suggesting that the diagnosis of dysfunctional labor should be based on the number of births. Another study focusing on the first phase of the labor curve and the rate of cervical dilation temporarily [29] found no significant differences between women who underwent TOLAC and those without previous C-sections. This research concludes that women undergoing TOLAC should be diagnosed with labor dysfunction under the same criteria as those without cesarean scars. Study investigating the labor patterns of primary parous women undergoing TOLAC without a history of vaginal birth and those experiencing natural labor [30] reported no differences in the first stage of labor. However, the median duration of the second stage of labor was slightly longer in the TOLAC group. The authors considered that they could not rule out the possibility that a higher incidence of basic labor complications contributed to the longer duration of labor observed. In this study, we included “history of vaginal birth” as one of the adjustment factors. Considering the lower proportion of women with a history of vaginal delivery in the TOLAC group, our result suggested that the observed impact on labor duration in the TOLAC group before adjustment could be partially attributed to the difference in vaginal birth history between the two groups.

Our study was novel in its examination of labor duration through the adjustment of participant profiles. However, this study had several limitations. First, the lack of data regarding the reasons for the previous C-section in the TOLAC group might have introduced selection bias. Nevertheless, we believe that by calculating propensity scores based on detailed maternal and fetal backgrounds and procedures, we were able to compare participants with similar backgrounds between the TOLAC and non-TOLAC groups after adjustment. Secondly, the onset of labor was based on self-reporting by pregnant women, and recall bias could not be eliminated. Although sensitivity analyses using interval censoring consistently showed similar trends, the issue was not entirely resolved. The reporting of the onset of labor by pregnant women was randomly erroneous without systematic bias, and the strength of the association may have been underestimated due to non-differential misclassification, biasing the results towards the null [31]. Third, this single-center study targeted a relatively stable population of pregnant women. The decision to attempt TOLAC is made by the women themselves after being informed of the risks and benefits. This may have influenced the outcomes of our study, as women who chose TOLAC might have had different characteristics or motivations compared to those who opted for ERCD. Fourth, this study utilized data collected over 10 years from January 1, 2012, to December 31, 2021. During this time, there might have been changes in clinical practice, such as advancements in obstetric care, updates to guidelines, or shifts in patient demographics. These potential changes could influence the management of TOLAC and the interpretation of the combined findings. Fifth, it was not possible to separately evaluate the first and second stages of labor.

## Conclusion

The impact of cesarean scars was limited, and the analysis after adjustment with IPTW suggested that other factors such as maternal physique and fetal characteristics had a more substantial influence.

### Electronic supplementary material

Below is the link to the electronic supplementary material.

Supplementary Material 1

Supplementary Material 2

## Data Availability

Availability of data and materials: The datasets generated and/or analyzed during the current study are available from the corresponding author upon reasonable request.
