# Supplementary material for: Evaluating the impact of a trial of labor after cesarean section on labor duration: a retrospective cohort study
Source: BMC Pregnancy Childbirth. 2024 Aug 15;24:542. doi: 10.1186/s12884-024-06744-0 (PMC11325700; doi:10.1186/s12884-024-06744-0)
Supplement: Supplementary file 1 — Supplementary Material 1 [file 12884_2024_6744_MOESM1_ESM.docx]

Supplemental table 1. Covariates adjusted for propensity score.

| **Characteristic** | **N** | **Non-TOLAC**  N = 170^1^ | **TOLAC**  N = 170^1^ | **Difference**^2^ | **95% CI**^23^ | **p-value**^4^ |
| --- | --- | --- | --- | --- | --- | --- |
| **Maternal age** | 340 |  |  | 0.04 | -0.17, 0.25 | 0.7 |
| Mean (SD) |  | 32 (5) | 32 (5) |  |  |  |
| **Maternal body mass index** | 340 |  |  | 0.06 | -0.15, 0.27 | 0.6 |
| Mean (SD) |  | 25.9 (3.9) | 25.6 (3.3) |  |  |  |
| **Maternal country of origin** | 340 |  |  | 0.07 | -0.14, 0.29 | 0.7 |
| Japanese |  | 158 (93%) | 161 (95%) |  |  |  |
| Other Nationality |  | 12 (7.1%) | 9 (5.3%) |  |  |  |
| **History of vaginal delivery** | 340 |  |  | 0.04 | -0.17, 0.25 | 0.8 |
| ( - ) |  | 126 (74%) | 129 (76%) |  |  |  |
| ( + ) |  | 44 (26%) | 41 (24%) |  |  |  |
| **Smoking** | 340 |  |  | 0.20 | -0.02, 0.41 | 0.10 |
| ( - ) |  | 158 (93%) | 148 (87%) |  |  |  |
| ( + ) |  | 12 (7.1%) | 22 (13%) |  |  |  |
| **Gestational diabetes mellitus** | 340 |  |  | 0.02 | -0.19, 0.23 | >0.9 |
| ( - ) |  | 152 (89%) | 153 (90%) |  |  |  |
| ( + ) |  | 18 (11%) | 17 (10%) |  |  |  |
| **Premature rupture of membranes** | 340 |  |  | 0.04 | -0.17, 0.25 | 0.8 |
| ( - ) |  | 121 (71%) | 118 (69%) |  |  |  |
| ( + ) |  | 49 (29%) | 52 (31%) |  |  |  |
| **Fetal sex** | 340 |  |  | 0.01 | -0.20, 0.22 | >0.9 |
| Female |  | 81 (48%) | 82 (48%) |  |  |  |
| Male |  | 89 (52%) | 88 (52%) |  |  |  |
| **Fetal birth weight** | 340 |  |  | 0.11 | -0.10, 0.32 | 0.3 |
| Mean (SD) |  | 3,124 (373) | 3,081 (416) |  |  |  |
| **Fetal position** | 340 |  |  | 0.11 | -0.10, 0.32 | >0.9 |
| Cephalic position |  | 170 (100%) | 169 (99%) |  |  |  |
| breech presentation |  | 0 (0%) | 1 (0.6%) |  |  |  |
| **Labor induction** | 340 |  |  | 0.06 | -0.15, 0.27 | 0.8 |
| ( - ) |  | 162 (95%) | 164 (96%) |  |  |  |
| ( + ) |  | 8 (4.7%) | 6 (3.5%) |  |  |  |
| **Labor analgesia** | 340 |  |  | 0.05 | -0.16, 0.26 | 0.8 |
| ( - ) |  | 160 (94%) | 158 (93%) |  |  |  |
| ( + ) |  | 10 (5.9%) | 12 (7.1%) |  |  |  |
| **Vacuum-assisted delivery** | 340 |  |  | 0.08 | -0.14, 0.29 | 0.6 |
| ( - ) |  | 154 (91%) | 150 (88%) |  |  |  |
| ( + ) |  | 16 (9.4%) | 20 (12%) |  |  |  |
| **Uterine fundal pressure** | 340 |  |  | 0.00 | -0.21, 0.21 |  |
| ( - ) |  | 170 (100%) | 170 (100%) |  |  |  |
| ( + ) |  | 0 (0%) | 0 (0%) |  |  |  |
| ^1^n (%) | | | | | | |
| ^2^Standardized Mean Difference | | | | | | |
| ^3^CI = Confidence Interval | | | | | | |
| ^4^Welch Two Sample t-test; Pearson's Chi-squared test | | | | | | |
