# Supplementary material for: Evaluating the impact of a trial of labor after cesarean section on labor duration: a retrospective cohort study
Source: BMC Pregnancy Childbirth. 2024 Aug 15;24:542. doi: 10.1186/s12884-024-06744-0 (PMC11325700; doi:10.1186/s12884-024-06744-0)
Supplement: Supplementary file 2 — Supplementary Material 2 [file 12884_2024_6744_MOESM2_ESM.docx]

Supplemental table 2. Results of sensitivity analysis using interval censoring.

| **Interval** | **HR^1^**  **(unweighted^3^)** | **95% CI^2^**  **(unweighted^3^)** | **p-value**  **(unweighted^3^)** | **HR^1^**  **(weighted^4^)** | **95% CI^2^**  **(weighted^4^)** | **p-value**  **(weighted^4^)** |
| --- | --- | --- | --- | --- | --- | --- |
| ±4 hours | 0.68 | 0.48 - 0.97 | 0.031 | 1.00 | 0,90 - 1.11 | 0.96 |
| ±8 hours | 0.45 | 0.24 - 0.83 | 0.011 | 1.05 | 0.87 - 1.27 | 0.63 |
| ±12 hours | 0.40 | 0.20 - 0.79 | 0.008 | 1.23 | 0.99 - 1.51 | 0.058 |
| ^1^ HR = Hazard Ratio  ^2^ CI = Confidence Interval  ^3^ Without inverse probability of treatment weighting  ^4^ With inverse probability of treatment weighting | | | | | | |
